# Inflammatory, procoagulant markers and HIV residual viremia in patients receiving protease inhibitor monotherapy or triple drug therapy: a cross-sectional study

**DOI:** 10.1186/1471-2334-14-379

**Published:** 2014-07-10

**Authors:** Miriam Estébanez, Natalia Stella-Ascariz, Jesús Mingorance, Ignacio Pérez-Valero, Jose Ignacio Bernardino, Francisco Xavier Zamora, Maria Luisa Montes, Juan Julián González-García, José Ramón Arribas

**Affiliations:** 1 HIV Unit, Internal Medicine Service, Hospital Universitario La Paz, IdiPAZ, Madrid, Spain; 2 Microbiology Service, Hospital Universitario La Paz, IdiPAZ, Madrid, Spain

**Keywords:** HIV, Monotherapy, Inflammation, Residual viremia

## Abstract

**Background:**

Protease inhibitor monotherapy is associated with more frequent episodes of viral rebounds above 50 copies/mL than triple therapy. Objective: To evaluate if, compared to triple-drug therapy, protease inhibitor monotherapy is associated with increased levels of inflammatory/procoagulant markers and more frequent plasma residual viremia detection.

**Methods:**

In this cross-sectional study, we included patients treated for ≥ 1 year with darunavir/ritonavir or lopinavir/ritonavir as monotherapy (n = 72) or with two nucleos(t)ides (n = 74). All samples were tested for CRP, IL-6, fibrinogen and D-dimer. Residual viremia was determined using an ultrasensitive qualitative nested-PCR of the HIV pol gene with a limit of detection of 1 copy of HIV-RNA.

**Results:**

We found no differences in levels of inflammatory/procoagulant markers or in the proportion of patients with plasma residual viremia detection by treatment group.

**Conclusion:**

The long-term treatment with protease inhibitor monotherapy in the setting of routine clinical practice is not associated with a higher prevalence of plasma residual viremia or more elevated inflammatory/procoagulant markers levels than triple drug therapy.

## Background

In clinical trials, protease inhibitor (PI) monotherapy (MT) has been effective in maintaining long-term viral suppression in the majority of patients [1]. However, MT is associated with more frequent episodes of viral rebounds above 50 copies/mL than triple therapy (TT). It has been suggested that episodes of low level viremia might lead to higher levels of inflammation and procoagulant markers such as interleukin-6 (IL-6), C-reactive protein (CRP) and D-dimer.

In the MONET clinical trial [2], virologically suppressed patients were randomized to darunavir/ritonavir as MT or as TT. In a subset of patients enrolled in MONET no differences were found in IL-6 and CRP levels between the MT and TT arms in stored samples at week 144 [3]. In MONET, residual plasma viremia (RV) below 50 copies/mL was not directly measured so it remains unknown if higher levels of inflammatory/procoagulant markers are correlated with the level of RV. In the present study we sought to investigate the relationship between PI MT, levels of inflammatory/procoagulant markers and RV.

## Methods

The present study is a subanalysis of a parent study comparing neurocognitive impairment in patients treated with protease inhibitor monotherapy or triple drug antiretroviral therapy. Details of this cohort and the recruitment flow-chart have been previously published [4]. We included patients who were currently receiving for ≥ 1 year lopinavir or darunavir as MT or as TT and had suppressed plasma viral load (<50 copies/ml) for at least one year. 179 patients on MT were selected, 41 patients rejected inclusion and 40 patients were screening failure. In TT group, 238 patients were selected. 49 patients rejected inclusion and 91 patients were screening failure. So, 98 patients were recruited in each group. After that, two patients on MT and three patients on TT were excluded due to HIV-1 RNA above 50 copies/ml at the initial study visit. Finally, we included 96 in the MT group and 95 patients in the TT group in the parent study. In the present study, we had available stored samples from 72 patients on MT and 74 patients on TT.

All samples were tested for CRP, IL-6, fibrinogen and D-dimer. Routine determination of viral load was done with Nuclisens EasyQ HIV-1 v2.0 (bioMeriéux, Marcy-l’Étoile, France). This technique quantifies the viral load above 20 copies/ml. At the same time-point, plasma RV was determined using an ultrasensitive qualitative nested-PCR of the HIV pol gene with a limit of detection of 1 copy of HIV-RNA. This method is based on the PCR used for single genome sequencing [5]. The local Ethics Committee for Clinical Research (La Paz University Hospital, Madrid) approved all the procedures. All participants provided written informed consent.

Sample characteristics were described using absolute and relative frequencies for categorical variables and median (IQR) for continuous variables. Chi-square test and Student’s t or the nonparametric Mann–Whitney U-test were used to compare baseline characteristics. We evaluated predictive factors of increased IL-6, CRP levels using a multivariate lineal regression model on log-transformed data. Explored factors were: age, sex, AIDS, CD4 (nadir/current), prior medical disease, hepatitis C (defined by HCV serum PCR), duration of viral suppression, detection of RV, blips (HIV RNA level > 50 copies/ml) in the last year, group of treatment, type of PI, triglycerides, total cholesterol/LDL ratio, total cholesterol/HDL ratio, HOMA index and use of statins. Variables with a *p*-value <0.1 in the univariate analysis, were retained in the model. All analyses were performed using the STATA statistical package (V.11.1, Stata Corporation, College Station, Texas, USA). All tests were 2-sided, p values <0.05 were considered significant.

## Results and discussion

Patients were predominantly male, with a median age of 46 years, and good immune status (Table 1). Compared to the TT group, the MT group showed significantly longer duration of suppressed HIV viremia (median 7.1 versus 4.7 years, p = 0.019). The median of time on PI monotherapy was 2.6 years (IQR 1.9-3.9).

**Table 1:** Demographics characteristics, HIV disease status and laboratory results

	**Triple therapy (n = 74)**	**Monotherapy (n = 72)**	**p-value**
Receiving darunavir/ritonavir. N (%)	19 (26)	27 (37.5)	0.124
Receiving abacavir. N (%)	12 (16.2)	NA	
Receiving tenofovir. N (%)	60 (81.0)	NA	
Receiving zidovudine. N (%)	2 (2.7)	NA	
Receiving lamivudine. N (%)	20 (27.0)	NA	
Receiving emtricitabine. N (%)	54 (72.9)	NA	
Male. N (%)	52 (70.3)	52 (72.2)	0.794
Age. Median (IQR)	44 (40–48)	47 (44–51)	0.002
Prior medical disease*. N (%)	27 (36.5)	36 (50)	0.099
Hepatitis C active. N (%)	18 (24.3)	14 (19.4)	0.932
AIDS. N (%)	47 (63.5)	44 (61.1)	0.764
CD4 nadir (cells/μL). Median (IQR)	141 (47–244)	172 (53–268)	0.376
Current CD4 (cells/μL). Median (IQR)	576 (417–805)	623 (457–834)	0.225
Years virologically suppressed.			
Median (IQR)	4.7 (2.9–8.9)	7.1 (4.5–8.9)	0.019
Monotherapy	NA	2.6 (1.9–3.9)	
Patients with a single blip **. N (%)	8 (10.8)	4 (5.5)	0.249
Detectable HIV RNA (Ultrasensitive assay). N (%)	33 (44.6)	28 (38.9)	0.484
HIV RNA 20–49 copies/ml. N (%)	12 (16.2)	5 (6.9)	0.081
Triglycerides (mg/dL). Median (IQR)	149 (111–196)	189 (137–248)	0.007
LDL Cholesterol. Median (IQR)	125 (107–153)	132 (110–156)	0.161
Total Cholesterol/HDL ratio. Median (IQR)	3.8 (3.3–4.7)	4.6 (3.7–5.6)	0.003
Total Cholesterol/LDL ratio. Median (IQR)	1.6 (1.5–1.8)	1.6 (1.5–1.8)	0.668
Receiving statins. N (%)	17 (25.4)	16 (24)	0.484
HOMA index. Median (IQR)	1.7 (1.0–2.7)	2.1 (1.3–3.4)	0.105
CRP level(mg/L).Median (IQR)	0.9 (0–2.4)	1.3 (0–2.8)	0.478
CRP ≥5 mg/L. N (%)	5 (6.8)	10 (14.5)	0.139
IL-6 (pg/ml).			
Median(IQR)	1.9 (1.2–3.4)	1.7 (1.3–3)	0.504
IL-6 ≥ 3 pg/ml. N (%)	17 (23)	18 (25)	0.774
Fibrinogen (mg/dl). Median (IQR)	386 (351–452)	398 (362–447)	0.372
D-dimer (ng/ml). Median (IQR)	131 (99–214)	137 (99–234)	0.769

Medical disease: hypertension, dyslipaemia, diabetes mellitus, ischemic heart disease, heart insufficiency, chronic renal failure, thyroid disorders and peripheral arterial disease*. Blip: detection of RNA-HIV > 50 copies/ml in the last year**. NA: non applicable.

We found no differences in levels of inflammatory/procoagulant markers between groups of treatment. Specifically we have not found differences in IL-6 levels ≥3 pg/mL and CRP levels ≥ 5 mg which have been associated with higher rates of progression to AIDS or death and an increased cardiovascular risk [6,7]. These results are comparable to those of the MONET trial [3]. In the multivariate analysis, the variables significantly associated with increased IL-6 level were male gender (p = 0.017), older age (p = 0.009) and active hepatitis C (p = 0.043) and the variable significantly associated to increased CRP levels was the HOMA index (p = 0.030). Hepatitis C coinfection was also associated with higher IL-6 level in the MONET trial [3]. Overall, there was a significant weak correlation between higher IL-6 levels and higher CRP levels (correlation coefficient = 0.35, p < 0.001). The detection of RV was similar in both groups of treatment (44.6% in TT group vs. 38.9% in MT group, p = 0.484). The percentage of patients with viral load 20–49 copies/ml was higher in triple therapy group (p = 0.081). There were no differences in inflammatory/procoagulant markers or detection of RV between patients receiving MT with Lopinavir/r or Darunavir/r.

We have found that the use of PI MT in routine clinical practice, compared to TT, is not associated to higher levels of inflammatory or procoagulant markers. Interestingly, as in prior studies [3,8] hepatitis C coinfection was significantly associated to higher IL-6 level.

In our study, plasma RV -detectable in nearly half of patients- was not more frequent in patients receiving MT. In the “Only Kaletra” pilot trial [9] the level of RV did not increase after switching from TT to lopinavir/ritonavir MT. In the MONET clinical trial the percentage of patients with HIV RNA <5 copies/mL remained constant over time without differences between MT and TT [10]. In the MONOI trial, among patients with viral load <50 copies/ml at week 48 there was no difference in the proportions of patients with HIV RNA < 1 copy/mL at week 48 [11]. Taken together these results do not suggest that MT is associated with more frequent detection of RV.

Our study did not show an association between detection of RV and higher levels of inflammatory/procoagulant markers. In the study by Chun et al. [12] RV was detected in 63% of virologically suppressed patients with no association with increased levels of inflammatory/procoagulant markers. In the FRAM cohort, there was little association of low-level viremia with levels of CRP, IL-6 and fibrinogen. Only in patients with viral loads above 10,000 HIV-RNA copies/mL there was an association with increased IL-6 levels [13].

Our study has the intrinsic limitations of cross-sectional analyses. We compared MT and TT in a selected group of patients, who have maintained long-term virological suppression and results cannot be extrapolated to other types of patients. However this limitation is inherent to the fact that MT is only indicated in adherent patients with suppressed viral load [14]. The time on viral suppression was significantly longer in the MT group, which could have decreased the possibility of detection of residual viremia in this group. However, the duration of viral suppression and the group the treatment were not significantly associated with increased levels of IL-6 and CRP in the multivariate analysis.

## Conclusion

In summary, our study suggests that long-term treatment with darunavir/ritonavir or lopinavir/ritonavir MT in the setting of routine clinical practice is not associated with a higher prevalence of plasma RV or more elevated inflammatory/procoagulant markers levels than triple drug therapy.

## Competing interests

The authors declare that they have no competing interests.

## Authors’ contributions

ME and JRA conceived of the study, and participated in its design, coordination and wrote the draft. NS and JM carried out the nested-PCR assays. IPV, JIB, FXZ and MLM participated in the design of the study, inclusion of patients and helped in the statistical analysis. JGG helped in the coordination and drafted of the manuscript. All authors read and approved the final manuscript.

## Pre-publication history

The pre-publication history for this paper can be accessed here:

http://www.biomedcentral.com/1471-2334/14/379/prepub
